# Peroxisome proliferator-activated receptor alpha is essential factor in enhanced macrophage immune function induced by angiotensin converting enzyme

**DOI:** 10.21203/rs.3.rs-4255086/v1

**Published:** 2024-04-22

**Authors:** Suguru Saito, DuoYao Cao, Ellen A. Bernstein, Anthony E. Jones, Amy Rios, Aoi O. Hoshi, Aleksandr B. Stotland, Erika E. Nishi, Tomohiro Shibata, Faizan Ahmed, Jennifer E. Van Eyk, Ajit Divakaruni, Zakir Khan, Kenneth E. Bernstein

**Affiliations:** 1 Department of Biomedical Sciences, Cedars-Sinai Medical Center, Los Angeles, CA 90048, USA; 2 Department of Molecular and Medical Pharmacology, University of California, Los Angeles, Los Angeles, CA 90095, USA.; 3 Graduate School of Comprehensive Human Science, University of Tsukuba, Tsukuba, Ibaraki 3058577, Japan; 4 Department of Cardiology, Smidt Heart Institute, Cedars-Sinai Medical Center, Los Angeles, CA, 90048, USA.; 5 Department of Physiology, São Paulo School of Medicine, Universidade Federal de São Paulo, Rua Botucatu, 862 terreo, Sao Paulo, 04023-062, Brazil; 6 Department of Pathology and Laboratory Medicine, Cedars-Sinai Medical Center, Los Angeles, CA 90048, USA; 7 Division of Gastroenterology, Cincinnati Children’s Hospital Medical Center, Cincinnati, OH 45229, USA

**Keywords:** angiotensin converting enzyme, macrophages, PPARα, anti-tumor immunity, bacterial clearance

## Abstract

An upregulation of angiotensin-converting enzyme (ACE) expression strengthens the immune activity of myeloid lineage cells as a natural functional regulation mechanism in our immunity. ACE10/10 mice, possessing increased ACE expression in macrophages, exhibit enhanced anti-tumor immunity and anti-bactericidal effects compared to those of wild type (WT) mice, while the detailed molecular mechanism has not been elucidated yet. In this report, we demonstrate that peroxisome proliferator-activated receptor alpha (PPARα) is a key molecule in the functional upregulation of macrophages induced by ACE. The expression of PPARα, a transcription factor regulating fatty acid metabolism-associated gene expressions, was upregulated in ACE-overexpressing macrophages. To pinpoint the role of PPARα in the enhanced immune function of ACE-overexpressing macrophages, we established a line with myeloid lineage-selective PPARα depletion employing the Lysozyme 2 (LysM)-Cre system based on ACE 10/10 mice (named A10-PPARα-Cre). Interestingly, A10-PPARα-Cre mice exhibited larger B16-F10-originated tumors than original ACE 10/10 mice. PPARα depletion impaired cytokine production and antigen-presenting activity in ACE-overexpressing macrophages, resulting in reduced tumor antigen-specific CD8+ T cell activity. Additionally, the anti-bactericidal effect was also impaired in A10-PPARα-Cre mice, resulting in similar bacterial colonization to WT mice in Methicillin-Resistant *Staphylococcus aureus* (MRSA) infection. PPARα depletion downregulated phagocytic activity and bacteria killing in ACE-overexpressing macrophages. Moreover, THP-1-ACE-derived macrophages, as a human model, expressing upregulated PPARα exhibited enhanced cytotoxicity against B16-F10 cells and MRSA killing. These activities were further enhanced by the PPARα agonist, WY 14643, while abolished by the antagonist, GW6471, in THP-1-ACE cells. Thus, PPARα is an indispensable molecule in ACE-dependent functional upregulation of macrophages in both mice and humans.

## Introduction

Macrophages play a critical role in the innate and adaptive immune response to infection and tumors (1, 2). They also are central to the pathogenesis of atherosclerosis, a chronic disease in which vascular lipid accumulation is in part due to macrophage dysfunction (3). Our group has studied the immune response of a mouse genetic model called ACE 10/10 in which monocytes and macrophages express increased amounts of angiotensin-converting enzyme (ACE) (4). Such mice have a profound increase of macrophage function in response to diverse immune challenges, including models of infection, tumor growth, atherosclerosis, and Alzheimer’s disease (5–11). While studying the mechanism by which ACE affects myeloid function, it became evident that increased ACE expression induced a remarkable change in the metabolism of these cells characterized by an increase in oxidative metabolism, increased lipid utilization via β oxidation, and increased cell content of adenosine triphosphate (ATP) (9, 10, 12). Importantly, more ACE expression was associated with increased expression of peroxisome proliferator activated receptor α (PPARα) (9, 10).

PPARα is a transcription factor that is recognized as a master regulator of cell lipid metabolism (13). With ligand stimulation, PPARα forms a dimer with the retinoid X receptor and then binds to PPAR response elements (PPREs) in nuclear DNA to stimulate transcription of dozens of genes that affect the cytoplasmic and mitochondrial metabolism of many different classes of lipids, including cholesterol, triglycerides, phospholipids, bile acids, and fatty acids (14). Hepatocytes produce PPARα and have served as the model system for investigating the effects of this protein (15). PPARα is also made by monocytes and macrophages where it regulates genes associated with metabolism, but it also affects other macrophage functions including reverse lipid transport (16, 17). However, the role of PPARα in myeloid immune function has not been studied in depth.

While increased expression of ACE by macrophages results in both a profound change in metabolism, and a marked increase of macrophage function, the exact relationship of these two processes is not fully understood. Specifically, a key question is whether the increased PPARα present in ACE expressing macrophages directly causes the increased immune response of these cells. To investigate this, we have created ACE 10/10 mice that also have a floxed PPARα gene and are transgenic for the LysM-Cre gene. The result of these modifications is an ACE 10/10 mouse retaining elevated macrophage ACE expression but in which there is selective reduction of myeloid PPARα expression. Here, we show that such mice have a marked reduction of the enhanced immune response present in ACE 10/10 mice. These data implicate PPARα as an important mediator of metabolism and of the increased immune function present in ACE 10/10 mice. Our results suggest that PPARα is a potential locus for manipulation to optimize and perhaps increase the immune response.

## Results

### Selective depletion of PPARα in ACE 10/10 mice

**Figure 1.**
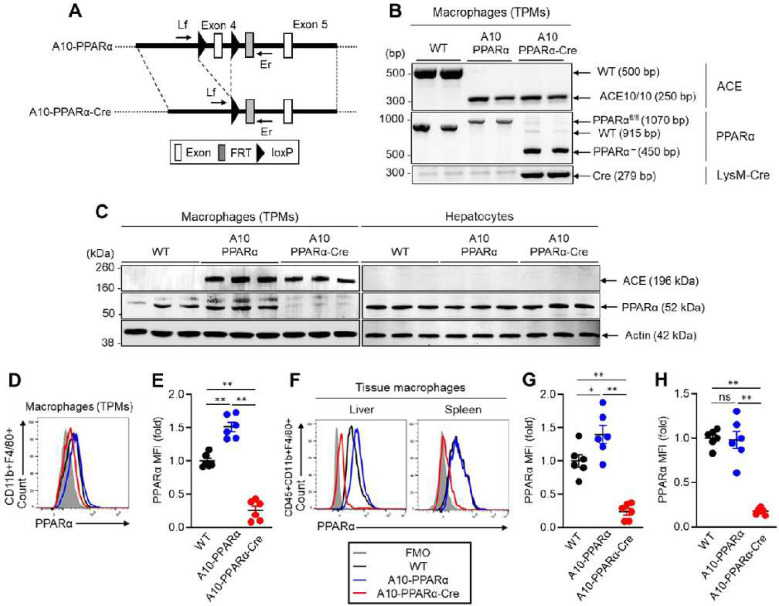
Genetic design of macrophage specific PPARα deletion in ACE10/10 mice. A) A10-PPRAα mice (floxed) were crossed with LysM-Cre mice to obtain A10-PPRAα-Cre mice (conditional KO). PPARα exon 4 was excised by the Cre-loxP system. To measure the depletion of exon 4, PCR was performed by using the primers Lf and Er (Supplement Table 1). B) Representative gel images of genotyping PCR for determination of WT, A10-PPARα and A10-PPRAα-Cre mice. C) PPARα expression in thioglycolate-elicited peritoneal macrophages (TPMs) and hepatocytes measured by Western blot. D, E) Representative histogram (D) and MFI values (fold change) (E) of PPARα expression in TPMs measured by flow cytometry. F-H) Representative histogram (F) and PPARα MFI values (fold change) in spleen (G) and liver (H) resident macrophages measured by flow cytometry. The cumulative data are shown as mean ± SEM values of six samples from two independent experiments. All MFI values are represented as fold changes (the average value of WT was used for a value equal to 1). One-way ANOVA was used to analyze data for significance. *p < 0.05 and **p < 0.001, ns is not significant.

The creation and characterization of ACE 10/10 and ACE knockout (KO) mice were previously described (5–12). Earlier study revealed that PPARα expression was elevated in thioglycolate elicited peritoneal macrophages (TPMs) from ACE 10/10 mice and reduced in equivalent cells from ACE KO mice as compared to wild type (WT) macrophages (9). Further, there was a direct correlation between PPARα expression in the TPMs of individual mice and levels of cell surface antigen presentation-related molecules (CD80, CD86, etc.), antigen uptake, phagocytic activity, and the ability to stimulate T cells (Supplemental Figure 1A-K). To further investigate the role of PPARα in ACE 10/10 macrophage function, breeding was used to create ACE 10/10 mice homozygous for the floxed PPARα gene described by Montag et al (Figure 1 A, B) (18). These mice are termed A10-PPARα. We also made A10-PPARα mice that were heterozygous for the LysM-Cre gene described by Clausen (19). These animals are termed A10-PPARα-Cre. The expectation was that A10-PPARα mice would phenocopy ACE 10/10 while the A10-PPARα-Cre mice would have low levels of myeloid cell PPARα due to myeloid specific Cre recombinase expression eliminating exon 4 in the PPARα cording region. Finally, we also selectively studied ACE 10/10 mice which were heterozygous for the LysM-Cre gene but have non-floxed PPARα genes (termed ACE 10/10-Cre mice) to confirm no background effect of heterozygous expression of the LysM gene.

Initial characterization used Western blot analysis to examine PPARα production by TPMs and by hepatocytes (Figure 1C). We also measured PPARα expression of TPMs and tissue resident macrophages (liver and spleen) by flow cytometry (Figure 1D–H). These screenings showed an 83% reduction of PPARα expression in macrophage from A10-PPARα-Cre mice as compared to A10-PPARα mice and about 75% reduction as compared to WT cells. In contrast, there was no appreciable change in hepatocyte expression of PPARα. We also measured PPARα expression in bone marrow (BM) isolated neutrophils and found no difference between A10-PPARα ± Cre recombinase expression (Supplemental Figure 2). The percentages of T cells, B cells, macrophages, monocytes, dendritic cells, and neutrophils were analyzed in spleen, liver, lymph nodes (LN), bone marrow (BM), and peripheral blood (PB). PPARα-depletion did not affect the frequency of these cells in the A10-PPARα-Cre mice compared to A10-PPARα mice and WT mice (Supplemental Figure 3A-E).

### Gene characterization of PPARα depleted macrophages

To further characterize the change in gene expression induced by the reduction of macrophage PPARα expression, TPMs of WT, A10-PPARα, and A10-PPARα-Cre mice were examined by bulk RNA sequencing. This analysis showed that the reduction of macrophage PPARα expression in A10-PPARα-Cre mice results in a marked change in the gene expression pattern. Specifically, comparing the number of genes in which there were differences in RNA expression between the three groups studied showed that a comparison of A10-PPARα vs. WT cells identified 2276 differences in gene expression, A10-PPARα vs. A10-PPARα-Cre found 1811 differences, while A10-PPARα-Cre vs. WT identified only 1499 differences (differences were defined as fold change (FC) ≥ 2 or FC ≤ 0.5 in gene expression, Supplemental Figure 4). This was the first suggestion that A10-PPARα-Cre macrophages more closely resembled WT cells rather than A10-PPARα cells.

**Figure 2.**
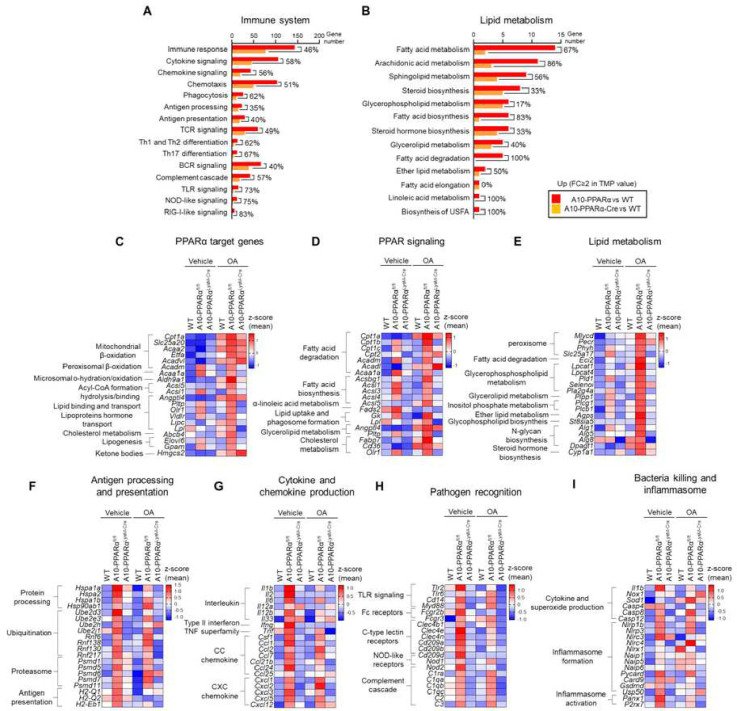
PPARα depletion alters the expression of immune response and lipid metabolism associated genes in macrophages. A, B) The number and percent difference in number of genes with ≥ 2-fold increased RNA expression in the comparison of A10-PPARα (red) and A10-PPRAα-Cre (orange) TPM vs. WT TPM. The classifications of gene categories (A: Immune system; B: Lipid metabolism) were determined using KEGG Term level 1. (C-I) Gene expression heat maps of individual PPARα target genes (C), PPAR signaling (D), Lipid metabolism (E), Antigen processing and presentation (F), Cytokine and chemokine production (G), Pathogen recognition (H), and Bacteria killing and inflammasome (I).

Two physiologic areas where there are large numbers of RNA expression differences are for genes affecting the immune system and genes that participate in lipid metabolism (Figure 2A, B, Supplemental Figure 5A, B). These figures show the number of genes different from WT cells compared to either A10-PPARα or A10-PPARα-Cre macrophages. In every sub-category of immune response or lipid metabolism, the A10-PPARα macrophages (high PPARα) have many more differences from WT cells than the A10-PPARα-Cre macrophages compared to WT. For example, among the genes that the KEGG pathway database assigns to ‘cytokine signaling’, the A10-PPARα macrophages expressed 143 genes at levels increased more than 2-fold the levels observed in WT cells, while A10-PPARα-Cre macrophages expressed only 77 genes different from WT, a reduction of 58%.

Because PPARα is known to regulate genes in response to lipid, we analyzed the gene expression changes of A10-PPARα, A10-PPARα-Cre, and WT macrophages in the presence and absence of oleic acid (OA), a mono-unsaturated omega-9 fatty acid. Heat maps show the relative expression levels of the 20 genes having the highest z-score among the genes assigned to the categories ‘PPARα target genes’, ‘PPAR signaling’, and ‘Lipid metabolism’ (Figure 2C–E). These data showed that, even under basal conditions, 71% of these genes are up-regulated in the A10-PPARα cells compared to WT with only 29% of genes that are reduced. In the presence of OA, this trend is exaggerated with 98% of genes being increased in A10-PPARα. In contrast, under basal conditions, only 43% of genes of the A10-PPARα-Cre cells are increased as compared to WT cell and 53% are decreased. Even after OA treatment, only 51% of genes are increased in A10-PPARα-Cre cells.

Figures 2F–I show expression levels of genes associated with antigen processing and presentation, cytokines/chemokines production, pathogen recognition, and bacterial killing/inflammasome. For each of these measures of the immune response, A10-PPARα macrophages expressed significantly more transcripts than either WT or A10-PPARα-Cre macrophages. In fact, all the heat map presentations make plain that the inability of the A10-PPARα-Cre macrophages to express PPARα is associated with a consistent reduction of RNA expression such that these cells resemble WT macrophages.

### Metabolism of PPARα depleted macrophages

**Figure 3.**
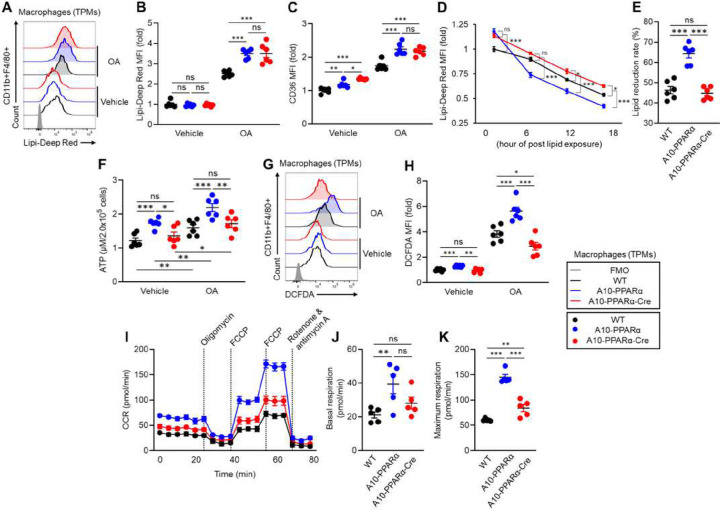
PPARα depletion alters lipid metabolism in ACE-overexpressing macrophages. TPMs were incubated with vehicle (ethanol) or OA (200 μM) at 37°C for 16 h. The intracellular lipid was stained with Lipi-Deep Red (LDR) and quantified by flow cytometry. A, B) Representative histograms and MFI values (fold change) of LDR signals in TPMs. C) MFI values (fold change) of CD36 expressions in TPMs measured by flow cytometry. D-E) TPMs were treated with OA for 16 h, washed and then placed in media for 6, 12 and 18 h. Intracellular lipid was stained with LDR and quantified by flow cytometry at each time point. D) Time dependent lipid reduction. E) Lipid reduction rate at 18 h. F-H) ATP and ROS production in TPMs. TPMs were incubated with control (ethanol) or OA (200 μM) at 37°C for 16 h. The ATP concentration in TPMs was measured by luminescence (F). The intracellular ROS levels of TPMs were assessed by DCFDA staining and flow cytometry. G, H) Representative histograms and MFI values (fold change) of DCFDA signals for ROS quantification. I-K) Real-time metabolic analysis of TPM by Seahorse. I) Transition of oxygen consumption rate (OCR) in TPMs during analysis. J, K) Basal respiration and maximal respiration of TPMs. The cumulative data are shown as mean ± SEM values of five to six samples from two independent experiments. All MFI values are represented as fold changes (the average value of WT was set as equal to 1). One-way ANOVA was used to analyze data for significance. *p < 0.05, **p < 0.01 and ***p < 0.01. ns is not significant.

To examine the functional effects of PPARα depletion, TPMs were cultured with or without OA and uptake of lipid was measured by staining the cells with the lipid specific dye Lipi-Deep Red (LDR) and then measuring intensity by flow analysis (Figure 3A, B). Without OA treatment, basal lipid content was similar between the three groups of TPMs. However, with OA, the TPMs from the A10-PPARα mice and A10-PPARα-Cre mice showed an equivalent increase in lipid uptake as compared to WT cells. In the same experiment, we also measured expression of the scavenger receptor CD36 (Figure 3C). These data mimicked uptake of lipid in that OA treatment increased CD36 expression in both the A10-PPARα and A10-PPARα-Cre cells as compared to WT cells. A different result was obtained when we examined the metabolism of lipid. For this experiment, TPMs from the three groups of mice were cultured with OA for 16 h and washed, then intracellular content of lipid was measured by flow cytometry with LDR staining at 6, 12 and 18 hours post lipid exposure. By comparing the slope of these curves and the final amount of intracellular lipid, we were able to estimate the ability of each population of macrophage to metabolize intracellular lipid (Figure 3D, E). As we previously reported, ACE 10/10 macrophages (equivalent here to A10-PPARα) have an enhanced ability to clear intracellular lipid as compared to WT cells (9). In contrast, the reduction of PPARα in A10-PPARα-Cre TPM resulted in a marked reduction in the rate in which macrophages can clear intracellular lipid.

We previously reported that ACE 10/10 macrophages contain more intracellular ATP and can produce more reactive oxygen species (ROS) then equivalent WT cells (9, 10, 12). As above, we tested TPMs from the three groups of mice cultured with and without OA for both ATP and ROS levels. This showed that the A10-PPARα cells contained more ATP than either WT or A10-PPARα-Cre TPM both with or without OA addition (Figure 3F). We found that ROS production is similar in the 3 groups of cells in the absence of OA, but with OA treatment, the A10-PPARα cells showed increased ROS production compared to WT. In contrast, ROS production in A10-PPARα-Cre cells was reduced even below WT levels (Figure 3G, H).

Finally, we measured basal oxidative metabolism of TPMs from the three groups of mice using the Seahorse analyzer. As we predicted based on the ATP data, the A10-PPARα cells had both a higher basal respiration (consumption of oxygen) and a higher maximum respiration than WT cells. In contrast, the loss of PPARα expression in A10-PPARα-Cre cells was associated with a basal respiration rate similar to WT cells and a maximal respiration rate that was significantly diminished from A10-PPARα though still was somewhat elevated compared to WT (Figure 3I–K).

We also performed metabolomics to determine the intracellular levels of several metabolites in TPMs. Whole metabolome analysis using a partial least squares-discriminant analysis (PLS-DA) plot showed that each of the three groups (A10-PPARα, A10-PPARα-Cre, and WT) had a distinct metabolic pattern with that of the A10-PPARα-Cre cells shifting toward that of WT cells (Supplemental Figure 6A). Additionally, some of the identified metabolites associated with mitochondrial oxidative metabolism were increased in A10-PPARα TPMs compared to both A10-PPARα-Cre and WT cells (Supplemental Figure 6B).

We also measured ATP, ROS production, and lipid uptake activity in TPMs from ACE 10/10-Cre mice that express LysM-Cre, but without PPRAα floxed genes. All results were comparable between ACE 10/10 and ACE 10/10-Cre TPMs (Supplemental Figure 7A-C) indicating that expression of cre recombinase (or modification of the *Lyz2* gene) was not the cause of the downregulation of metabolic activity in A10-PPARα-Cre TPMs.

### Reduced tumor resistance of A10-PPARα-Cre mice

**Figure 4.**
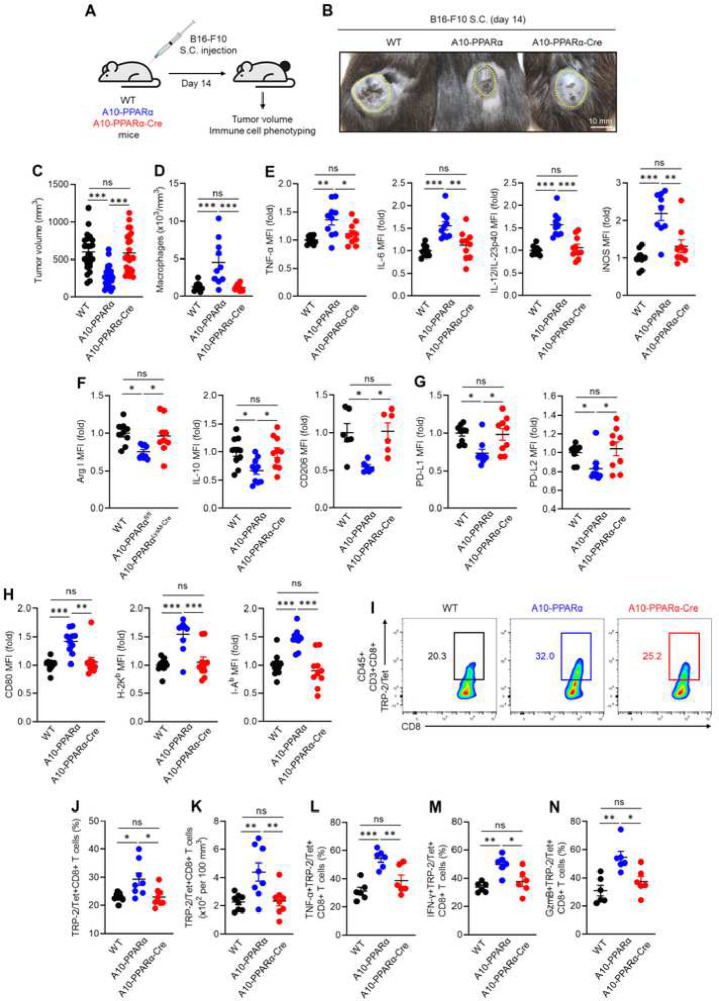
PPARα depletion impairs anti-tumor activity of A10-PPARα-Cre mice. A) Experimental design of murine B16-F10 tumor model. The mice received a subcutaneous (s.c.) injection of B16-F10 cells (100 μL of 1.0×10^7^/mL in PBS). The tumor volumes were measured and immunological activities of intratumor (IT) macrophages and CD8^+^ T cells were analyzed by flow cytometry at day 14 post tumor inoculation. B) Representative pictures of tumors. C) Tumor volumes. D) Number of macrophages infiltrating the tumors. E-H) Functional marker expression by IT macrophages. MFI values (fold change) of M1 markers (TNF-α, IL-6, IL-12/IL-23p40 and iNOS) (E), M2 markers (arginase 1 (Arg 1), IL-10, and CD206) (F), co-inhibitory molecules (PD-L1 and PD-L2) (G), and antigen presentation related molecules (CD80, H-2K^b^ and I-A^b^) (H). I-N) Functional characterization of IT CD8^+^ T cells. I) Representative plots of TRP-2/Tetramer (Tet)^+^CD8^+^ T cells. J, K) Percentages and cell numbers of TRP-2/Tet^+^CD8^+^ T cells. L-N) Percentages of TNF-α^+^, IFN-γ^+^, or GzmB^+^TRP-2/Tet^+^CD8^+^ T cells. The cumulative data are shown as mean ± SEM values of six to ten samples from two or three independent experiments. All MFI values are represented as fold changes (the average value of WT was used for base=1). One-way ANOVA was used to analyze data for significant differences. *p < 0.05, **p < 0.01, and ***p < 0.001. ns is not significant.

To assess the functional role of PPARα in the immune response, we challenged WT, A10-PPARα, and A10-PPARα-Cre mice with subcutaneous injection of murine melanoma B16-F10 cells (6). On day 14 after tumor inoculation, mice were sacrificed, and the excised tumors were analyzed. We published previously that ACE 10/10 mice have a substantially better anti-tumor response than WT and display smaller tumors at sacrifice (6). This same pattern was seen with the A10-PPARα mice; tumors were significantly smaller than in WT mice. In contrast, the tumor size was significantly larger in A10-PPARα-Cre mice and was similar to tumor growth in WT mice (Figure 4A–C).

During B16-F10 challenge to ACE 10/10 mice, many of the characteristics associated with pro-inflammatory M1 macrophages are increased (6). This pattern was reproduced in A10-PPARα mice. Specifically, as compared to WT animals, A10-PPARα mice have more macrophages within the tumor and these intratumor (IT) macrophages expressed increased levels of cytokines such as tumor necrosis factor α (TNF-α), interleukin-6 (IL-6), IL-12/IL-23p40, and inducible nitric oxide synthase (iNOS) (Figure 4D, E). In terms of M2 associated markers, IT macrophages from A10-PPARα expressed lower levels of arginase I (Arg I), IL-10, and CD206 than WT cells (Figure 4F). Further, the A10-PPARα cells expressed lower levels of the immunosuppressive molecules programmed death-ligand 1 (PD-L1) and PD-L2 (Figure 4G). Remarkably, for all these measures of increased inflammatory activity, the A10-PPARα-Cre macrophages have a phenotype equivalent to WT cells.

As indicated, the A10-PPARα mice have IT macrophages that appear more inflammatory than WT. These cells expressed increased CD80, H-2K^b^ (MHC class I) and I-A^b^ (MHC class II) indicating the potential for better antigen presentation to T cells (Figure 4H). This appears to result in a better CD8^+^ T cell tumor response as indicated by higher frequency and number of IT CD8^+^ T cells with specificity for the melanoma tumor antigen tyrosinase related protein-2 (TRP-2) (TRP-2/Tet^+^CD8^+^ T cells) (Figure 4I–K) and higher expression of TNF-α, IFN-γ, and granzyme B (GzmB) in TRP-2/Tet^+^CD8^+^ T cells (Figure 4L–N). Yet again, the A10-PPARα-Cre mice are essentially equivalent to WT mice for all these measures of immune response.

**Figure 5.**
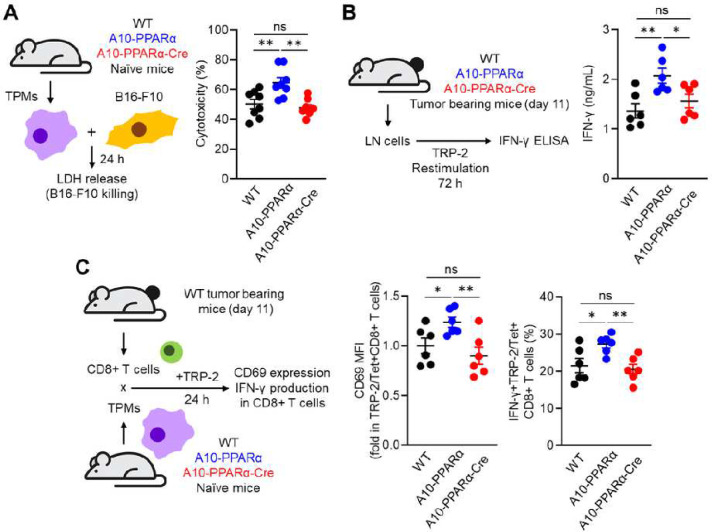
PPARα depletion attenuates tumor killing and antigen presenting ability of ACEover expressing macrophages. A) *In vitro* tumor killing assay. B16-F10 cells and TPMs prepared from WT, A10-PPARα or A10-PPARα-Cre naive mice (no tumor) were mixed at a 1:1 ratio and incubated at 37°C for 24 h. The LDH concentrations in the cultures were measured to calculate the percentages of tumor cells killed by TPMs. B) *In vitro* antigen re-stimulation assay. Inguinal lymph node (iLN) cells were isolated from tumor bearing mice 11 days after tumor inoculation and were re-stimulated with TRP-2 peptide (100 μg/mL) at 37°C for 72 h. IFN-γ concentrations in the cultured medium were measured by ELISA. C) *In vitro* antigen presentation assay. CD8^+^ T cells were isolated from the iLNs of tumor bearing WT mice 11 days after tumor inoculation. TPMs were also prepared from naive WT, A10-PPARα or A10-PPARα-Cre mice and co-cultured with the CD8^+^ T cells in the presence of TRP-2 peptide (100 μg/mL) at 37°C for 24 h. The expression (MFI) of CD69 by CD8^+^ T cells and the percentages of IFN-γ^+^CD8^+^ T cells were measured by flow cytometry. The cumulative data are shown as mean ± SEM of six samples from two independent experiments. All MFI values are represented as fold changes (the average value of WT was set equal to 1). One-way ANOVA was used to analyze data for significant differences. *p < 0.05 and **p < 0.01. ns is not significant.

We have also performed *in vitro* functional assays of macrophages. The first of these is the ability of macrophages to kill B16-F10 tumor cells*.* For this experiment, TPMs from either WT, A10-PPARα, or A10-PPARα-Cre mice were mixed with B16-F10 tumor cells. Direct tumor killing activity of TPMs was assessed by measuring lactate dehydrogenase (LDH) release. This showed a pattern consistent with previous results where tumor killing observed in A10-PPARα cells was greater than that observed by macrophages from WT mice, but that cells from A10-PPARα-Cre mice showed killing at WT levels (Figure 5A). We also performed an *in vitro* T cell restimulation assay to estimate the abundance of tumor antigen specific T cells. B16-F10 tumor cells were inoculated into the 3 groups of mice and inguinal lymph node (iLN) cells were isolated at day 11 post tumor inoculation. The iLN cells were re-stimulated with TRP-2 peptide and tumor antigen specific T cell response was assessed by INFγ production. Again, the iLN cells from the A10-PPARα mice produced more IFN-γ in responds to TRP-2 stimulation than WT cells, while the response of A10-PPARα-Cre iLN cells was equivalent to WT (Figure 5B).

Finally, we performed an antigen presentation assay using TPMs and tumor sensitized CD8^+^ T cells. CD8^+^ T cells were isolated from the iLNs of tumor-bearing WT mice after 11 days of B16-F10 inoculation. These CD8^+^ T cells were mixed with TPMs from either WT, A10-PPARα, or A10-PPARα-Cre mice in the presence of the TRP-2 peptide. After incubation for 24 hours, we measured the expression of CD69 and IFN-γ in TRP-2/Tet^+^CD8^+^ T cells. Again, the TPMs originating from A10-PPARα mice showed the highest CD8^+^ T cell response by enhanced antigen presentation. The antigen presenting activities of either WT or A10-PPARα-Cre TPMs were equivalent and less than that observed with the A10-PPARα cells (Figure 5C). Thus, as measured in several different fashions, the selective reduction of PPARα expression in macrophages consistently reduced the immune response of A10-PPARα mice to a state equivalent to WT mice.

We have also separately analyzed the background effect of the LysM-Cre allele in the phenotype of ACE 10/10 mice using ACE 10/10-Cre mice. Analysis of B16-F10 tumor size in such mice showed no effect of LysM-Cre expression; these mice developed tumors equivalent in size to ACE 10/10 mice (Supplemental Figure 7D). We also measured IT macrophage function and the frequency of TRP-2/Tet^+^CD8^+^ T cells, none of which were different in the ACE 10/10-Cre mice compared to ACE 10/10 mice (Supplemental Figure 7E-I). These controls indicate that it is the lack of PPARα in the A10-PPARα-Cre mice that is affecting the anti-tumor response.

### Reduced bacterial resistance of A10-PPARα-Cre mice

**Figure 6.**
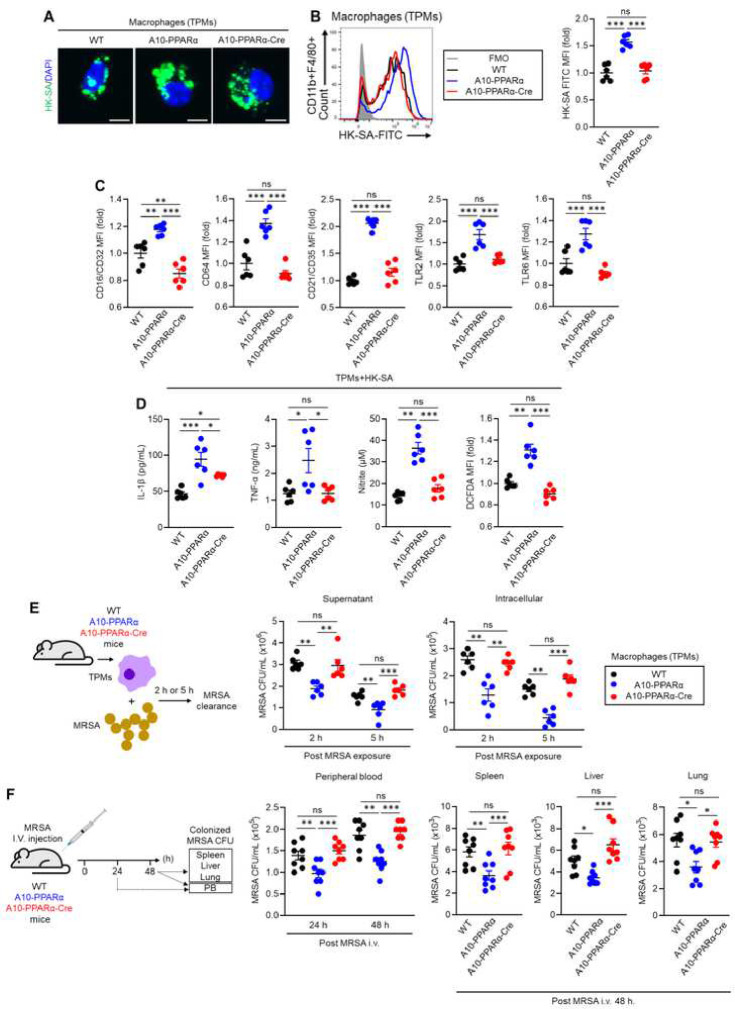
PPARα depletion impairs anti-bacterial immune response of A10-PPARα-Cre mice. A, B) *In vitro* phagocytosis assay. TPMs were incubated with FITC labeled heat killed *S. aureus* (HK-SA-FITC) at 37°C for 2 h. Bacterial phagocytosis was analyzed by fluorescence microscope and flow cytometry. Representative images of the incorporated HK-SA-FITC (green spots) in TPMs (bar=10 μm) (A), representative histograms and MFI values (fold change) of incorporated HK-SA-FITC signals in TPMs (B). C) TPM receptor expression. Representative histograms and MFI values (fold change) of CD16/CD32, CD64, CR1/2, TLR2 and TLR6 are shown. D) *In vitro* TPMs stimulation assay. TPMs were stimulated with HK-SA at 37°C for 24 h and the concentrations of IL-1β, TNF-α and nitrite in the cultured medium were measured by ELISA and Griess assay. The ROS productions in TPMs were measured by flow cytometry with DCFDA staining. E) *In vitro* MRSA killing assay. TPMs (1.0×10^6^/mL) were incubated with MRSA (1.0×10^7^ CFU/mL, MOI=1:10) for 2 h or 5 h, and the number of live MRSA in the supernatant and within the TPM were quantitated as colony forming units (CFUs). F) *In vivo* MRSA infection. Mice received an i.v. injection of live MRSA (100 μL of 1.0×10^9^ CFU/mL in PBS). After 24 h or 48 h, MRSA CFUs were measured for peripheral blood (PB). The CFUs were also measured for spleen, liver, and lung at 48 h (per 100 mg of tissue). The cumulative data are shown as mean ± SEM values of six or eight samples from two or three independent experiments. All MFI values are presented as fold changes (the average value of WT was set as 1). One-way ANOVA was used to analyze data for significant differences. *p < 0.05, **p < 0.01 and ***p < 0.01. ns is not significant.

To investigate an immune challenge different from B16-F10 melanoma, we studied the ability of the three groups of mice to respond to challenge from methicillin-resistant *Staphylococcus aureus* (MRSA)*.* In the first experiment, we performed an *in vitro* phagocytosis assay using TPMs from the three groups of mice. The TPMs were mixed with FITC-labeled heat-killed *S. aureus* (HK-SA-FITC) for 2 hours, then phagocytotic activity was assessed by flow cytometry. Again, the pattern was typical of the previous data in that A10-PPARα TPMs captured more bacteria than WT or A10-PPARα-Cre TPMs (Figure 6A). Further, the TPMs from WT, A10-PPARα, and A10-PPARα-Cre mice showed significant differences in surface receptor expression important in anti-bacterial activity. Specifically, TPMs from A10-PPARα mice expressed increased basal levels of CD16/CD32 (FcγRII/III), CD64 (FcγRI), CD21/CD35 (complement receptor (CR) 1/2), Toll-like receptor (TLR) 2, and TLR6 compared to WT and A10-PPARα-Cre cells (Figure 6B). We note that these evaluations of protein expression match the RNA expression presented in Figure 2H. WT and A10-PPARα-Cre TPMs expressed equal levels of these surface receptors except for CD16/CD32 where cells from A10-PPARα-Cre mice expressed significantly lower levels than even WT cells.

Cytokines and chemical mediator production was measured in TPMs exposed to HK-SA. TPMs from the three groups of mice incubated with HK-SA for 24 hours showed a consistent pattern where the A10-PPARα cells expressed greater quantities of IL-1β, TNF-α, ROS, and nitrite. The TPMs from the A10-PPARα-Cre mice expressed lesser amounts that were equivalent to WT cells except for IL-1β where the level of expression in the A10-PPARα-Cre TPMs was intermediate to that of WT and the A10-PPARα mice (Figure 6C).

To directly assess macrophage killing of live bacteria, TPMs from the three groups of mice were mixed *in vitro* with MRSA for 2 or 5 hours, the macrophages were then separated by centrifugation, and the number of colony forming units (CFUs) in the supernatant and in lysed macrophages was assessed (Figure 6D). The observed pattern was again characterized by TPMs from the A10-PPARα mice being most efficient at killing the MRSA while cells from the other two groups were both equivalent and lesser in terms of efficacy of bacterial killing.

As a final experiment to measure *in vivo* resistance to bacterial infection, mice from the three groups were injected i.v. with MRSA and bacterial CFUs were measured in the peripheral blood (PB) at 24 hr and in PB, liver, spleen, and lung at 48 hours. For the solid organs, tissue was homogenized in PBS and bacteria measured per 100 mg of tissue (Figure 6E). In these assays, the results were similar to what was previously observed: samples from A10-PPARα mice showed significantly fewer MRSA CFUs than the samples from the other two groups. Again, these last two groups were not significantly different. Finally, the TPMs from ACE 10/10-Cre mice (PPARα gene not floxed) showed similar activity against bacteria as compared to ACE 10/10 (Supplemental Figure 7J-M). Thus, myeloid cell PPARα depletion in A10-PPARα-Cre mice critically impairs both macrophage anti-tumor and anti-bacterial response.

### PPARα is a key regulator of ACE-dependent functional up-regulation in human macrophage-like cells

**Figure 7.**
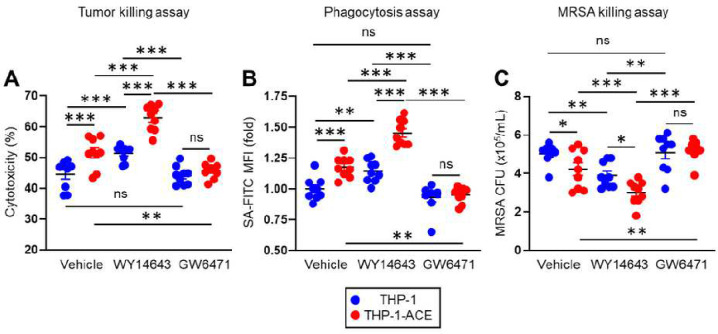
PPARα regulates ACE-mediated functional behavior of human macrophage-like cells. THP-1 and THP-1-ACE cells were differentiated to macrophage-like cells by treating with PMA (20 ng/mL) for 72 h. A) *In vitro* tumor killing assay. BT549 cells and macrophage-like cells were mixed at a 1:1 ratio and incubated at 37°C for 24 h. The LDH concentrations in the culture media were measured to calculate killing of tumor cells. B) *In vitro* phagocytosis assay. Macrophage-like cells were incubated with FITC labeled *S. aureus* (SA-FITC) for 2 h at 37°C at which point bacterial phagocytosis was quantified by flow cytometry. MFI values are presented as fold change where the average value of WT cells was set as 1. C) *In vitro* MRSA killing assay. Macrophage-like cells were incubated with MRSA (MOI=1:30) at 37°C for 5 h. The number of live intracellular MRSA was then determined by CFU analysis. The cumulative data are shown as mean ± SEM values of nine samples from three independent experiments. One-way ANOVA was used to analyze data for significant differences. *p < 0.05, **p < 0.01 and ***p < 0.01. ns is not significant.

To investigate the role of PPARα in ACE-mediated enhancement of human immune cells, we performed *in vitro* functional assays using the human monocytic cell lines THP-1 and THP-1 genetically altered to stably over express ACE (termed THP-1-ACE,). These cells were studied after differentiation to macrophage-like cells [9]. Additionally, the assays were performed in the presence or absence of the PPARα agonist WY14643 or the PPARα antagonist GW6741 [9, 20]. An *in vitro* tumor killing assay found that THP-1-ACE had significantly increased cytotoxicity against BT549 cells, a human breast cancer cell line, compared to THP-1 cells. WY14643 treatment increased the activity of both THP-1 and THP-1-ACE cells, and also increased the difference between the two groups. In contrast, the difference was abolished by the PPARα antagonist GW6471 (Figure 7A). The phagocytic activity of THP-1-ACE for fluorescently labelled *S. aureus* was also significantly increased as compared to THP-1. Again, WY14643 treatment increased the phagocytic activity of both cell types and the difference between the two groups, while GW6471 decreased the activity of THP-1-ACE cells resulting in it being equivalent to THP-1 (Figure 7B). Finally, we performed a bacterial killing assay by mixing cells and MRSA at a 1:30 ratio and then, after a 5 hr incubation at 37° C, measured the number of live intracellular bacteria. This showed that the killing of MRSA by THP-1-ACE cells was significantly greater than THP-1 cells. Killing was increased by WY14643 and impaired by GW6471 (Figure 7C). Thus, over expression of ACE enhances human macrophage function similar to observations in mouse macrophages, and PPARα consistently emerges as a necessary factor in regulating this functional modification.

## Discussion

Discovering ways to increase the immune response is important both from the perspective of understanding immune system function and from a practical desire to increase immunity against tumors and serious infection. Such increased immunity is observed in the ACE 10/10 mice, animals in which the ACE gene was placed under the control of the myeloid specific *c-fms* promoter resulting in increased ACE protein production by monocytes and macrophages. What was observed in several previous studies is that ACE 10/10 mice have a highly effective immune response to a variety of immune challenges, including tumors, infection, and models of chronic diseases such as atherosclerosis and Alzheimer’s disease (5–11). Despite having an immune response that is far more effective than WT, these animals have no evidence of autoimmunity. The phenotype of ACE 10/10 mice is directly due to increased catalytic activity of ACE since ACE inhibitors reduce the immune behavior of these animals to that of WT mice treated similarly (5–11). Further, a different line of mice having increased ACE expression by neutrophils demonstrated significantly increased neutrophil effectiveness, both *in vitro* and *in vivo*, against several different pathogenic bacteria (20, 21). While studies of the immune function of ACE in humans are far less extensive than the studies in mice, there is evidence that blocking ACE activity in humans (and mice) reduces neutrophil activity against bacteria (21). Thus, it is potentially very important to understand specifically how ACE activity enhances myeloid cell immune function.

Recently it was discovered that ACE activity markedly changes the metabolism of myeloid cells expressing the enzyme (9, 10, 12). With increased ACE, there is a significant increase in oxidative metabolism, particularly of lipids, with increased concentrations of cell ATP. These metabolic changes are so striking that they ineluctably lead to the hypothesis that this is what underpins the increased functionality of macrophages expressing increased ACE.

PPARα was first discovered in 1990 as a ligand receptor capable of inducing hepatic peroxisome proliferation (22). Substantial subsequent analysis, particular in the hepatic function of PPARα, has established that this transcription factor is a central regulator of lipid metabolism (22, 23). Of the three PPARs known (α, γ and β/δ), in macrophages there is higher expression of PPARγ than PPARα (24–26) and, perhaps because of this, there is not extensive study of the functional effects of PPARα in macrophages. However, some immune aspects in PPARα have been studied in different cell types and systems. Study of PPARα global knockout (KO) mice, which have an increase in cytokine expression in some instances, has suggested an anti-inflammatory role for this protein (26, 27). In another study, PPARα-KO mice showed increased helper CD4^+^ T cell type 17 (Th17) generation resulting in increased pathology of murine experimental autoimmune encephalomyelitis (28). Another study reported that inhibition of PPARα activity restored the anti-tumor effect of dendritic cells (29).

Our interest in PPARα came from finding that this protein was elevated in ACE 10/10 macrophages under both basal conditions and after exposure to lipid, and that several known targets of PPARα, such as carnitine palmitoyl transferase 1A (CPT1A), CPT1B, CPT2, acyl-CoA dehydrogenase long chain (ACADL), CD36, and ATP-binding cassette transporter 1 (ABCA1), were elevated in these same cells (9, 10). In contrast, no such elevation was noted for PPARγ or PPARδ. Lipid tracing using ^13^C-OA showed that ACE 10/10 macrophages metabolized lipid far more avidly than WT cells (9). Further, the higher oxidative metabolism and increased ATP levels in these cells appeared to support increased macrophage functional capacity (9, 10, 12). That PPARα stimulates fatty acid β-oxidation within mitochondria, and that this provides abundant ATP, led us to create the A10-PPARα-Cre mice to precisely evaluate the role of this master lipid regulator in the exceptional immune response of ACE 10/10 mice. Indeed, one of the critical findings of our study is that ATP levels in A10-PPARα-Cre macrophages were reduced to levels present in WT cells. Whether ATP is the full explanation or, more likely, that the global change in metabolism present in ACE 10/10 macrophages underpins the increased function remains to be determined. But what is clear is that all our data support the idea that elevated PPARα is required for the increased immune response of the ACE 10/10 mice, whether this is evaluated in terms of gene expression, analysis of macrophage metabolism, resistance to B16-F10 melanoma, ability to activate T cells, or protection from MRSA. The behavior of A10-PPARα-Cre mice is that of WT mice. Stated differently: no PPARα equals no exceptional immune response.

Our data raise two questions. One is the role of PPARα in WT macrophages. A10-PPARα-Cre mice have PPARα expression less than 20% the levels found in A10-PPARα cells, but they also have levels that average less than 30% those of WT cells (see Figure 1). The finding that A10-PPARα-Cre macrophages and A10-PPARα-Cre mice are so consistently similar to WT suggests that in WT animals, PPARα does not play a major role in macrophage immune function. This conclusion is supported by the data in Figure 7 showing that treatment of THP-1 cells (the control cells for this experiment) with the PPARα inhibitor GW6471 has very little effort on THP-1 function, at least as measured by cytotoxicity, phagocytosis, and bacterial killing.

Our data raise a second much more important question: whether forcing increased PPARα expression or activity in macrophages would lead to increased immune function. This was tested *in vitro* by treating THP-1 and THP-1-ACE cells with the PPARα selective ligand WY14643. As noted above, WY14643 increased the immune function of both cell types. This is important to investigate further since selective stimulators of PPARα are available for humans (30, 31).

In summary, previous study of ACE 10/10 mice have documented the remarkable increase in mouse immune function achieved by increasing macrophage expression of ACE. An important component of this is increased monocytic expression of PPARα which is now revealed as important in regulating macrophage function.

## Materials and Methods

### Reagents and antibodies

Oleic acid (OA), BSA (fraction V, fatty acid free), collagenase type II, phorbol 12-myristate 13-acetate (PMA) and ionomycin were purchased from Millipore-Sigma (Burlington, MA, USA). 2’,7’-dichlorodihydrofluorescein diacetate (H_2_DCFDA) and 10x red blood cell (RBC) lysis buffer (muti species), fluorescein isothiocyanate (FITC)-labeled *Escherichia coli* (E. coli) K-12 strain, and FITC-labeled heat-killed *Staphylococcus aureus* (HK-SA) were purchased from Thermo Fisher Scientific (Waltham, MA, USA). Macrophage colony-stimulating factor (M-CSF) was purchased from PeproTech (Westlake Village, CA, USA). Heat-killed *Staphylococcus aureus* (HK-SA) was purchased from InvivoGen (San Diego, CA, USA). CellTiter-Glo^®^ 2.0 was purchased from Promega (Madison, WI, USA). Lipi-Deep Red was purchased from Dojindo Laboratories (Kumamoto, Japan). Monoclonal antibodies (mAbs) and the tetramer used for flow cytometry were shown in Supplemental Table 1. The Abs used for western blotting were shown in Supplemental Table 2 and 3.

### Mice

To make ACE10/10-PPARα-floxed mice, WT PPARα-floxed mice [original made by Dr. Walter Wahli (18), University of Lausanne] were obtained from Dr. Mingyu Liang, Medical College of Wisconsin. These mice were crossed with ACE 10/10 mice. The A10-PPARα mice were further crossed with LysM-Cre mice (Jackson Labs strain #4781) (19) that was a gift of Dr. Gislaine Martins, Cedars-Sinai Medical Center. Eventually, we obtained ACE10/10-PPARα-LysM-Cre (A10-PPARα-Cre). The genetic design of the mice was represented in Figure 1A. Genotyping PCR was performed by using the primers shown in Supplemental Table 4. All mice were bred with 12 h day/night cycles and were allowed free access to food and water. Gender and age-matched adult mice (8–16 weeks) were used for each experiment. All animal experimental protocols were reviewed and approved by the Animal Welfare Committee Cedars-Sinai Medical Center (#8780).

### Murine melanoma model

A murine melanoma model was established by following the method reported in a previous publication (6). Briefly, B16-F10 cells (1.0×10^6^ cells in 100 μL of PBS) were subcutaneously (s.c.) injected on the back skin of mice by using a 1 mL syringe with a 25G needle. The tumor volume was measured at day 14 by following a formula; V= (L × W^2^) × 0.52 (V: tumor volume, L>W). Finally, the mice were sacrificed at day 14 for immunological analysis. The excised tumor was studied for intratumor (IT) macrophage and T cell analysis.

### Preparation of thioglycolate-elicited peritoneal macrophages (TPMs)

The mice received an intraperitoneal (i.p.) injection of 3 mL of thioglycolate and the infiltrated leukocytes were harvested from the peritoneum at day 4 (between 84 to 96 h) post injection. The cells were seeded into 100 mm cell culture dishes in RPMI complete medium (RPMI1640 supplemented with 10% of fetal bovine serum (FBS), 100 U/mL of penicillin and 100 μg/mL of streptomycin) and incubated at 37° C for 3 h. The adherent cells, which are enriched TPMs, were washed with PBS three times and then detached by gentle scraping in PBS/2% FBS. The purity of TPMs was assessed by flow cytometry. The samples with CD11b+F4/80^+^ > 90% was used for subsequent experiments.

### Gene expression profiling

TPMs were treated with vehicle (ethanol) or OA (200 μM) in RPMI1640 medium supplemented with 1% of bovine serum albumin (BSA, fatty-acid free), 100 U/mL of penicillin, 100 μg/mL of streptomycin and macrophage colony-stimulating factor (M-CSF, 10 ng/mL) at 37° C for 48 h, The cells were washed with PBS twice, then used for total RNA isolation by using the RNeasy Plus Mini Kit (Venlo, Netherlands) following the product manual. The RNA samples with A260/280=1.8 to 2.0 were subjected to RNA sequencing. The RNA sequencing was performed by BGI (https://www.bgi.com/global) and the data was analyzed by Dr. Tom (https://biosys.bgi.com/#/report/login). Kyoto Encyclopedia of Genes and Genomes (KEGG) database (https://www.genome.jp/kegg/) was also used for analysis to enrich the gene clusters in the specific pathway and biological responses.

### Flow cytometry

Flow cytometry analysis was performed by using Cytek NL-3000 (Cytek Bioscience, Fremont, CA, USA). For extracellular marker staining, the samples were stained with fluorochrome-conjugated mAb or tetramer in the presence of anti-CD16/CD32 mAb for blocking of Fc gamma Receptor (FcγR) II/III at 4°C for 30 min. Intracellular and intranuclear staining were performed by using the BD Cytofix/Cytoperm^™^ Fixation/Permeabilization Kit (BD Bioscience, Franklin Lakes, NJ, USA) and the True-Nuclear Transcription Factor Buffer Set (BioLegend, San Diego, CA, USA), respectively. For intracellular staining, the extracellular-stained cells were fixed at 4°C for 20 min followed by staining of intracellular targets at 4°C for 30 min. For intranuclear staining, the extracellular-stained cells were fixed at room temperature (RT) for 60 min followed by intranuclear staining at RT for 30 min. The samples studied for cytokine detection in T cells were stimulated with PMA (100 ng/mL) and ionomycin (250 ng/mL) in the presence of GolgiStop^™^ (1 μg/mL, BD Bioscience) at 37°C for 5 h. The mAbs and tetramer used in the flow cytometry analyses were described in Supplemental Table 1. The samples were analyzed using flow cytometer by following the gating strategies represented in Supplemental Figure 8. The data were analyzed by FlowJo 10 (BD Bioscience).

### Western Blotting

For protein isolation, the cells were treated with RIPA buffer supplemented with protease inhibitor cocktail (1:100 dilution) and ethylenediaminetetraacetic acid (EDTA, 5 mM). The samples were sonicated at 4°C and stored at −20°C for overnight followed by centrifugation at 12,000 *g* for 15 min. The supernatant was collected as extracted protein sample. The protein concentration was determined by BCA. For loading sample preparation, the proteins were diluted with 5 x sodium dodecyl sulfate (SDS) sample buffer followed by boiling at 95°C for 5 min. The proteins were separated by SDS-polyacrylamide gel electrophoresis (SDS-PAGE) in 4–12% gradient gels (Thermo Fisher Scientific) followed by transfer onto polyvinylidene fluoride (PVDF) membranes. The membranes were blocked in blocking buffer at RT for 60 min, then treated with primary Ab at 4°C overnight. After washed with Tris-buffered saline containing 0.1% Tween 20 (TBST), membranes were treated with secondary Ab at RT for 60 min. The protein bands were visualized using an Odyssey CLx Infrared Imaging System (Li-Cor, St, Lincoln, NE, USA).

### Cell isolation from tumor

The tumor was excised from the mice back skin and briefly washed with PBS. The tumor was chopped with scissors and mechanically crushed on a 70 μm cell strainer in RPMI complete medium. The (IT) intratumor cells were collected by centrifugation at 300 *g* for 5 min, then the cells were digested with collagenase (1 mg/mL) at 37°C for 15 min with gently shaking. After being washed with RPMI complete medium, the cells were collected by centrifugation at 300 *g* for 5 min. The cells were used as tumor isolated cells for subsequent analysis.

### Primary cell isolation

Spleen and lymph nodes (LNs) were mechanically crushed on a 70 μm cell strainer in RPMI complete medium, then the cells were collected by centrifugation at 300 *g* for 5 min. The cells were treated with red blood cell (RBC) lysis buffer at RT for 10 min, then were washed with RPMI complete medium. After centrifugation at 300 *g* for 5 min, the precipitated cells were used as splenocytes and LN-isolated cells, respectively. Peripheral blood (PB) was treated with RBC lysis buffer at RT for 10 min followed by washing with PBS. After centrifugation at 300 *g* for 5 min, the precipitated cells were used as PB leukocytes. Bone marrow (BM) cells were isolated from femurs and tibias. The cells were flushed from the bones using 10 mL syringe with 27G needle in RPMI complete medium. The cells were collected by centrifugation at 300 *g* for 5 min, then treated with RBC lysis buffer at RT for 10 min. After washing with PBS, the cells were collected by centrifugation at 300 *g* for 5 min. The precipitated cells were used as BM-isolated cells. Hepatocytes and hepatic leukocytes were isolated from whole liver. The liver was excised from PBS-perfused mouse and chopped and mechanically crashed on a 70 μm cell strainer in RPMI complete medium. The cells were collected by centrifugation at 300 *g* for 5 min and the precipitated cells were re-suspended in 35% Percoll (diluted with PBS) for density gradient separation by centrifugation at 600 *g* for 20 min without rapid acceleration and external braking. After centrifugation, the top layer was collected as hepatocytes and precipitated cells were further treated with RBC lysis buffer at RT for 10 min. The samples were washed with RPMI complete medium and centrifuged at 300 *g* for 5 min. The precipitated cells were used as hepatic leukocytes.

### Metabolic assay of macrophages

TPMs (1.0×10^6^/mL) were cultured with vehicle (ethanol) or OA (200 μM) in RPMI 1640 medium supplemented with 1% BSA (fatty-acid free), 100 U/mL penicillin, 100 μg/mL streptomycin and M-CSF (10 ng/mL) at 37°C for 24 h. For the adenosine triphosphate (ATP) assay, the TPMs were washed with PBS, then treated with CellTiter-Glo^®^ 2.0 reagent at RT for 15 min followed by reading the luminescence intensity by microplate reader (FLUOstar Omega, BGM LABTECH, Ortenberg, Germany). The ATP concentration was determined by standard curve method. For detection of reactive oxygen species (ROS), the TPMs were treated with H_2_DCFDA (5 μM) at 37°C for 60 min followed by flow cytometry analysis. Alternatively, fresh TPMs were used for real-time cell metabolic assay using Seahorse cell analyzed (Agilent, Santa Clara, CA, USA).

### Fatty acid uptake and consumption assay

For the fatty acid uptake assay, TPMs (1.0×10^6^/mL) were cultured with vehicle (ethanol) or OA (200 μM) in RPMI 1640 medium supplemented with 1% BSA (fatty-acid free), 100 U/mL penicillin, 100 μg/mL streptomycin and M-CSF (10 ng/mL) at 37°C for 16 h. After washed with PBS, the TPMs were stained with Lipi-Deep Red (LDR, 1:200 dilution) at 37° C for 2 h. The lipid droplet (LD) content in the TPMs was analyzed by flow cytometry. To investigate the fatty acid consumption rate, the TPMs were treated with OA following the protocol of the fatty acid uptake assay. After 16 h of fatty acid treatment, the TPMs were washed with PBS and maintained in RPMI complete medium supplemented with M-CSF (10 ng/mL) until analysis. The TPMs were harvested at the indicated time points and stained with LDR (1:200) at 37°C for 2 h followed by flow cytometry analysis. The fatty acid consumption rate was calculated by following the formula: fatty acid consumption rate (%) equals (LDR MFI ^0 h^ −LDR MFI^18 h^)/LDR MFI ^0 h^ × 100.

### Real-time metabolic assay

TPMs (1.5×10^5^/100 μL) were seeded on Cell-Tak (Corning, Corning, NY, USA) coated Seahorse XF96 plates (Agilent Technologies, Santa Clara, CA, USA). All analyses were performed in RPMI1640 medium. Oligomycin (2 μM), FCCP (Carbonyl cyanide-p-trifluoromethoxyphenylhydrazone, 500 nm) and rotenone (200 nM) with antimycin A (1 μM) were added to the wells, and respiratory oxygen consumption rate was measured by Seahorse XF96 analyzer (Agilent Technologies). Metabolic parameters were calculated by following method as described in previous report (12).

### Tumor killing assay

TPMs (1.0×10^6^/mL) and B16-F10 cells (1.0×10^6^/mL) were mixed (at a ratio 1:1) in RPMI complete medium at 37° C for 24 h. Some cultures were prepared with TPMs or B16-F10 cells only for controls. The culture medium was harvested and stored at −80°C until use. The macrophage tumor cytotoxicity assay measured lactate dehydrogenase (LDH) release from B16-F10 cells. The abundance of released LDH was assessed by absorbance at 490 nm (background) and 680 nm (target) using the Pierce LDH Cytotoxicity Assay Kit (Thermo Fisher Scientific). The cytotoxicity was calculated by following the formula: % Cytotoxicity = (Co-culture’s LDH activity – spontaneous B16-F10 death LDH activity)/(Maximum B16-F10 LDH activity – Spontaneous B16-F10 LDH activity) × 100.

### In vitro T cell re-stimulation assay

The cells were isolated from inguinal lymph nodes (iLNs) of B16-F10 inoculated mice (day 11). The iLN cells (3.0×10^6^/mL) were re-stimulated with TRP-2 (100 μg/mL) at 37°C for 72 h. The IFN-γ concentration in the cultured medium was measured by ELISA.

### In vitro antigen presentation assay

CD8^+^ T cells were isolated from iLNs of B16-F10 inoculated WT mice (day 11). TPMs were prepared from WT, A10-PPARα or A10-PPARα-Cre mice. The CD8^+^ (5.0×10^6^/mL) and TPMs (1.0×10^6^/mL) were co-cultured in the presence of TRP-2 (100μg/mL) at 37°C for 24 h. The CD69 expression and IFN-γ production in TRP-2/Tet^+^CD8^+^ T cells were analyzed by flow cytometry.

### Phagocytosis assay

TPMs (1.0×10^7^/mL) were incubated FITC labeled *S. aureus* (SA-FIC; 25 μg/mL) in RPMI complete medium at 37°C for 2 h. The macrophages were washed with PBS and were analyzed by flow cytometry. The fluorescence signal (mean fluorescence intensity; MFI) originating from intracellular incorporated bacteria was used for assessing phagocytosis activity in the TPMs.

### Macrophage stimulation assay

TPMs (1.0×10^6^/mL) were seeded in 12 or 96 well plats with RPMI complete medium. The cells were treated with vehicle (PBS) or HK-SA (1.0×10^7^ CFU/mL) at 37°C for 24 h. After incubation, the cultured medium was harvested and stored at −80°C until use. The cytokine and nitric oxide (NO) concentrations in the cultured medium were measured by ELISA and the Griess assay, respectively. ROS production was measured by in flow cytometry with H_2_DCFDA staining.

### Methicillin-resistance Staphylococcus aureus (MRSA) infection

MRSA (USA300) was obtained from the ATCC (Manassas, VA, USA) and cultured by following a method provided by ATCC. Briefly, the frozen glycerol stock was thawed on ice and bacterial suspension was transferred to tryptic soy broth (TSB) medium. The culture was incubated at 37°C for overnight with shaking, then the grown bacteria were further cultured in TSB medium at 1:100 dilution at 37°C for 6–8 hours. The colony forming unit (CFU) was determined by standard curve method. The required number of bacteria were washed with PBS and collected by centrifugation at 10,000 *g* for 1 min. The collected bacteria were resuspended in PBS or RPMI complete medium for adjusting the concentration.

### In vitro MRSA killing assay

TPMs (1.0×10^6^/mL) were mixed with MRSA (1.0×10^7^ CFU/mL) in RPMI complete medium. The samples were incubated at 37°C for 2 or 5 h. The samples were first centrifuged at 300 *g* for 5 min, then the supernatants were collected, and the precipitated cells were washed with PBS. The cells were again collected by centrifugation at 300 *g* for 5 min, then treated with PBS/0.1% Triton X-100 at RT for 15 min to lyse the cells. The supernatant samples and cell lysates were used for determination of MRSA CFUs by seeding on a TSB agar plate. The plates were incubated at 37°C for overnight, then MRSA CFUs were determined in the samples.

### MRSA infection

MRSA infection was performed by following a method described in previous reports (20, 21). Briefly, the MRSA was washed and resuspended in PBS at 1.0×10^9^ CFU/mL concentration. The mice received an intravenous (i.v.) injection of MRSA (100 μl of suspension) through the retro orbital sinus. Blood samples were collected at 24 h and 48 h post infection. The tissue samples (spleen, liver, and lung) were collected at 48 h post infection. Before organ extraction, the mice were perfused with PBS. The tissues were washed with PBS and chopped and homogenized in PBS. The samples were serially diluted with PBS and seeded on TSB plates. The plates were incubated at 37°C for overnight, then MRAS CFUs were determined in the samples.

### Cytokine and nitric oxide measurement

Cytokine concentration in the sample was measured by ELISA kit (R&D systems, Minneapolis, MN, USA) for each target. Nitric oxide (NO) concentrations in the samples were measured by the Griess Reagent System (Promega). All procedures followed the product manual.

### THP-1 cell culture, differentiation, and functional assay

THP-1 or THP-1-ACE cells were cultured in RPMI complete medium and passaged every 5–6 days. For differentiation to macrophage-like cells, cells were cultured in RPMI complete medium supplemented with PMA (20 ng/mL) at 37°C for 72 h. Differentiated status was confirmed by the elevated expressions of CD11b and CD14 compared to undifferentiated cells. To measure *in vitro* tumor killing, the differentiated cells (1.0×10^6^/mL) were mixed with BT549 cells (1.0×10^6^/mL) at a ratio of 1:1 in RPMI complete medium supplemented with vehicle (DMSO), WY14643 (10 μM), or GW6471 (10 μM) at 37°C for 24 h. Some cultures were prepared with differentiated cells or BT549 cells only as controls. Differentiated cell killing of tumor cells was assessed by lactate dehydrogenase (LDH) release from the BT549 cells and cytotoxicity was calculated using the method described above. For the *in vitro phagocytosis assay,* the differentiated cells were initially cultured in RPMI complete medium supplemented with vehicle (DMSO), WY14643 (10 μM), or GW6471 (10 μM) at 37°C for 1618 h before being incubated with SA-FITC (50 μg/mL) at 37°C for 2 h. Cells were then washed with PBS and analyzed by flow cytometry; the MFI value originating from intracellular bacteria was used to assess phagocytic activity. To measure intracellular killing of MRSA, the differentiated cells were first treated with WY14643 or GW6471 as described above. The macrophage-like cells (1.0×10^6^/mL) were then mixed with MRSA (3.0×10^7^ CFU/mL) at 37°C for 5 h, washed, and lysed. Viable intracellular MRSA were then quantified by bacterial colony formation.

### Statistics

Student t-test and one-way analysis of variance (ANOVA) were used to analyze the data for significant differences. Values of *p* < 0.05, *p* < 0.01, and *p* < 0.001 were regarded as significant.
